# Surgical revascularization as a procedure to prevent neurological complications in children with moyamoya syndrome associated with neurofibromatosis I: a single institution case series

**DOI:** 10.1007/s00381-024-06304-z

**Published:** 2024-02-06

**Authors:** Alberto Morello, Marcello Scala, Irene Schiavetti, Maria Cristina Diana, Mariasavina Severino, Domenico Tortora, Gianluca Piatelli, Marco Pavanello

**Affiliations:** 1 Department of Neurosurgery, IRCCS Istituto Giannina Gaslini, Genoa, Italy; 2 Department of Neuroscience, Neurosurgery Unit, Rita Levi Montalcini”, “Città Della Salute e della Scienza” University Hospital, University of Turin, Turin, Italy; 3 Department of Neurosciences, Genetics, Maternal and Child Health, University of Genoa, Rehabilitation, Genoa, Ophthalmology Italy; 4 Medical Genetics Unit, IRCCS Istituto Giannina Gaslini, Genoa, Italy; 5 Department of Health Sciences, University of Genoa, Genoa, Italy; 6 Pediatric Neurology and Muscular Diseases Unit, IRCCS Istituto Giannina Gaslini, Genoa, Italy; 7 Neuroradiology Unit, IRCCS Istituto Giannina Gaslini, Genoa, Italy

**Keywords:** Moyamoya disease, NF1, Cerebral stenosis, Revascularization, Bypass, Anastomosis

## Abstract

**Background:**

The optimal timing and surgical approach for surgical revascularization in patients with moyamoya syndrome (MMS) associated with neurofibromatosis type I (NF1) remain so far elusive. We aimed to compare the long-term clinical, radiological, and cognitive effects of different revascularization procedures in a pediatric cohort of NF1-associated MMS.

**Methods:**

We reviewed the clinical, radiological, and surgical data of 26 patients with NF1-associated MMS diagnosed at our institution between 2012 and 2022, at the clinical onset and last follow-up.

**Results:**

Indirect bypasses were performed in 12/26 patients (57.1%), while combined direct and indirect procedures in 9/26 subjects (42.9%); 5 patients did not undergo surgery. Through logistic regression analysis, pathological Wechsler Intelligence Scale for Children (WISC) at onset was found to be associated with symptom improvement at 1-year follow up (*p* = 0.006). No significant differences were found in long-term neurocognitive outcome and stroke rate in patients receiving combined or indirect bypass (*p* > 0.05).

**Conclusions:**

Currently, whether combined or indirect bypass should be considered the treatment of choice in pediatric patients with NF1-associated MMS remains unclear, as well as the optimal time approach. In our series, no significant differences were found in long-term neurocognitive outcome and stroke rate between patients treated with either of these two approaches. Clinical evidence supports the crucial role of early diagnosis and surgical revascularization in subjects with MMS-associated NF1, even in case of mildly symptomatic vasculopathy. This allows to achieve a good long-term outcome with improved intellectual function and prevention of stroke and seizure in these patients.

**Supplementary Information:**

The online version contains supplementary material available at 10.1007/s00381-024-06304-z.

## Background

The diagnosis of neurofibromatosis type 1 (NF1) is based on the clinical criteria established by the National Institutes of Health (NIH) Consensus Development Conference [1]. In this condition, there is a large spectrum of central nervous system manifestations, including learning disability, mental retardation, seizures, attention deficit with hyperkinesia disorder, optic nerve glioma, and extra-optic pathways tumors [2]. Recent advances in the understanding of genotype–phenotype correlation are emerging concerning the association of cerebral vasculopathies in NF1. Vascular dysplasia is becoming increasingly emphasized as a feature of NF1 with an estimated frequency between 2 and 6% [3, 4]. The exact pathogenesis of cerebral stenosis and moyamoya syndrome (MMS) in patients with NF1 has not been elucidated yet [2]. RNF213 gene mutations have been associated with MMD, but a recent analysis showed no direct involvement of NF213 variants in the pathogenesis of moyamoya syndrome (MMS) in pediatric patients [5, 6]. The main hypothesis is that the loss of neurofibromin, the protein encoded by the NF1 gene, in vascular endothelial cells may be responsible for the excessive proliferation of vascular smooth muscle cells causing stenosis of the carotid arteries [7, 5].

Clinical manifestations of NF1 vasculopathy may stem from stenosis or occlusion of the vessels resulting in cerebral or visceral infarcts, aneurysms resulting in hemorrhage, or arteriovenous fistulae [8]. In patients with NF1, the prevalence of MMS is estimated at around 0.6% [9]. However, other authors reported a higher prevalence (3–6%), as most of the patients are often asymptomatic in the first stages of the disease [10]; on the other hand, an increased mortality has been reported in patients with NF1 and vasculopathy [11]. Patients with MMS generally become symptomatic because of ischemic complications, leading to transient ischemic events, seizures, motor and sensory symptoms, and neurocognitive deficits. The cognitive impairment is another important aspect to consider in patients with MMS. Indeed, on the basis of the natural history of NF1, approximately 80% of children develop deficits in one or more cognitive functions [12]. Currently, there is no known medical treatment capable of reversing the progression of MMS. However, anticoagulants/antiplatelet agents and vasodilators are employed with the purpose of slowing the progression of the vasculopathy.

Revascularization surgery has been shown to be a relatively safe and effective treatment for MMS. Revascularization prior to significant damage has been shown to improve cognition and quality of life, prevent neurocognitive decline if performed early, and modulate the natural history of the disease [13, 14]. The revascularization surgical approaches for MMS utilize branches of the preserved external carotid artery vasculature as donor vessels re-routed to augment blood flow to the ischemic territory of the affected internal carotid artery. This can be achieved either by direct or indirect revascularization methods. Direct revascularization involves direct vessel-to-vessel anastomosis of a branch of the external carotid artery, usually the superficial temporal artery to the internal carotid artery, or a more distal branch, usually the middle cerebral artery [15]. The indirect method involves placing a vascularized pedicle graft from the external carotid artery over the surface of the brain to promote angiogenesis [16]. Based on the tissue used, the main surgical techniques used are as follows: superficial temporal artery-middle cerebral artery (STA-MCA) bypass, encephalo-myo-synangiosis (EMS), encephalo-duro-synangiosis (EDAS) with dural graft, encephalo-duro-arterio-myo-synangiosis (EDAMS), encephalo-duro-myo-arterio-pericranio-synangiosis (EDAMPS), encephalo-duro-arterio-pericranio-synangiosis (EDAPS) [17].

Despite the evidence of a protective effect of surgical intervention in preventing poor cognitive outcome, especially if performed early in the disease course, there is no consensus about the ideal timing for revascularization surgery in NF1. Moreover, there are no randomized clinical trials in the literature comparing different revascularization techniques in children with NF1-associated MMS. Therefore, the surgical management of these cases is still performed on a case-based manner. The aim of our study is to compare the long-term clinical, radiological, and cognitive outcomes of children with NF1-associated MMS treated with different revascularization procedures, providing insights on technical features and timing of interventions.

## Methods

This is a retrospective observational study conducted on patients with NF1-associated MMS diagnosed at Gaslini Children’s Hospital between 2012 and 2022. Written informed consent was waived from the internal ethical committee due to the retrospective nature of the study. The study was conducted according to ethical standards laid down in the 1964 Declaration of Helsinki and its later amendments, following the Strengthening the Reporting of Observational Studies in Epidemiology (STROBE) guidelines.

Basic demographics and clinical data were extracted from electronic medical records. Data about neurological signs and symptoms, cognitive status, and epilepsy were collected at presentation at 1/2/5-year follow-up. Information on other organ involvement and genetic data were reported when available. Neurosurgical data regarding the type and timing of surgical revascularization were reviewed by two pediatric neurosurgeons (A.M. and M.P.). Cognitive impairment was studied by qualified neuropsychologists in each patient through the international scale: Wechsler Intelligence Scale for Children (WISC).

Brain MRI studies were performed on 1.5 T or 3 T magnetic resonance scanners with different protocols, all including an axial diffusion weighted imaging (DWI), axial and coronal T2-weighted images, 2D or 3D T1-weighted images, 2D or 3D FLAIR images, intracranial arterial MR angiography (MRA), and brain MR perfusion studies with contrast (T2* DSC) and/or without contrast with arterial spin labeling (ASL) techniques. MR images were inspected in consensus by two expert and pediatric neuroradiologists (M.S. and D.T.) for the presence, type, and site of cerebral arteriopathies, cerebral tumors, neurofibromas, and focal areas of signal intensity (FASI).

## Statistic

Statistical analysis was conducted using the Statistical Package for Social Sciences (SPSS) version 24.0. Continuous variables were presented as mean ± standard deviation (SD), while categorical variables were presented as counts with percentages. The effect of clinical data on the 1-year outcomes (presence or absence of symptoms) was evaluated through logistic regression analysis. The findings are displayed as odds ratios (OR) alongside their respective 95% confidence intervals, and a *p* value < 0.05 was considered statistically significant.

## Results

Twenty-six (26) children with NF1-assicated MMS were enrolled in the study (15 females, 57.7%). The age at diagnosis ranged from 1 to 23 years (mean 8.0 ± 5.06 SD). Thirteen children were asymptomatic (12/26, 46.2%), while the most common symptoms were headache (5/26, 19.2%) and focal neurological deficits (5/26, 19.2%). Two patients (7.7%) and one patient (3.8%) have manifested seizure and intellectual disability, respectively. Two patients have experienced other symptoms (7.7%). Baseline characteristics are shown in Table 1.

**Table 1:** Baseline characteristics

**Descriptive** ***N* = 26**		
Sex, females		15 (57.7%)
Age, years (DS)		8.0 ± 5.06
Ischemic infarct at onset (radiological)		12 (46.2%)
Artery: MCA		23 (88.5%)
Artery: ACA		7 (26.9%)
Artery: ICA		13 (50.0%)
Artery: PCA		8 (30.8%)
Vascular involvement side	Right	5 (19.2%)
	Left	8 (30.8%)
	Bilateral	13 (50.0%)
Ivy signs		15 (57.7%)
Delayed ATT (*N* = 23)		21 (91.3%)
Reduced CBF (*N* = 23)		4 (17.4%)
Side perfusion alteration (*N* = 21)	Unilateral	10 (47.6%)
	Bilateral	11 (52.4%)
Tumor		10 (38.5%)
Tumor type	Optic pathway glioma	7 (70.0%)
	Extra-OPG	2 (20.0%)
	OPG and extra-OPG	1 (10.0%)
Clinical score at onset	Asymptomatic	12 (46.2%)
	At least 1 symptom	14 (53.8%)
Clinical score at follow-up	Asymptomatic	12 (46.2%)
	1 symptom	11 (42.3%)
	2 symptoms	2 (7.7%)
	3 symptoms	1 (3.8%)
Initial symptom: headache		5 (19.2%)
Initial symptom: focal neurological deficits		5 (19.2%)
Initial symptom: seizure		2 (7.7%)
Initial symptom: intellectual disability		1 (3.8%)
Initial symptom: other		2 (7.7%)
Cognitive onset (pathologic WISC)	Normal	16 (61.5%)
	Pathological	10 (38.5%)
Radiological follow-up duration, years		6.09 ± 4.32
MMD progression (*N* = 25)		16 (64.0%)
Perfusion improved (*N* = 24)		18 (75.0%)
Additional ischemic infarcts (radiological, not clinical) at the last follow-up (*N* = 24)		4 (16.7%)
CT		5 (19.2%)
RT		3 (11.5%)
Surgery tumor		4 (15.4%)
Medical therapy		24 (92.3%)
Surgery		21 (80.8%)
Surgery type (*N* = 21)	Indirect	12 (57.1%)
	Combined	9 (42.9%)
Technique (*N* = 21)	STA-MCA + EMS + dural inversion	6 (28.6%)
	STA-MCA + EMS	2 (9.5%)
	EDAS/EDAMPS	3 (14.3%)
	EDAS	3 (14.3%)
	EDAMPS	3 (14.3%)
	EDAPS	1 (4.8%)
	Burr hole (other center)	1 (4.8%)
	EDAS + EMS	1 (4.8%)
	STA-MCA e EMS left + EDAMPS right	1 (4.8%)
Associated vasculopathies		2 (7.7%)
Type of vasculopathy	PFO	1
	RAS	1
Presence of symptoms at 1 year		4 (15.4%)

*DS* deviation standard, *MCA* middle cerebral artery, *ACA* anterior cerebral artery, *ICA* internal carotid artery, *PCA* posterior cerebral artery, *ATT* arterial transit delay, *CBF* cerebral blood flow, *OPG* optic nerve glioma, *WIS*C Wechsler Intelligence Scale for Children, *MMD* moyamoya disease, *CT* chemotherapy, *RT* radiotherapy, *symptoms* headache, seizure, intellectual disability, focal neurological deficits, *STA-MCA* superficial temporal artery-middle cerebral artery, *EMS* encephalo-myo-synangiosis, *EDAS* encephalo-duro-synangiosis, *EDAMS* encephalo-duro-arterio-myo-synangiosis, *EDAMPS* encephalo-duro-myo-arterio-pericranio-synangiosis, *PFO* patent foramen ovale, *RAS* renal artery stenosis

### Imaging analysis

At the first MRI examination, the anterior circulation was involved in 25/26 (96.1%) cases with additional posterior cerebral artery stenosis in 7/26 subjects (26.9%). One subject had isolated posterior MMS (3.8%). Overall, the middle cerebral artery (MCA) was affected in 23 cases (88.5%), the internal carotid artery (ICA) in 13 (50%), the anterior cerebral artery (ACA) in 7 cases (26.9%), and the posterior cerebral artery (PCA) in 8 patients (30.8%). Bilateral MMS was noted in 13/26 (50%) cases. Arterial ischemic infarcts were detected in 12 subjects (46.1%). The “ivy sign” on fluid-attenuated inversion recovery (FLAIR) or post-contrast T1-weighted images was found in 15 patients (15/26, 57.7%). Brain MR post-contrast perfusion studies were available in 23 cases and revealed delayed arterial transit time (ATT) in 21 cases (21/23, 91.3%), while reduced cerebral blood flow (CBF) was reduced in 4 subjects (4/23, 17,4%). Seven patients had an associated brain tumor (10/26, 38.4%).

At the last follow-up, performed on average after 6 years (range 1–17 years), additional ischemic lesions were detected in 4 cases. At brain MR perfusion, the ATT delay improved in 18/23 (78.2%) cases, while one subject showed worsening of perfusion parameters (1/23, 4%).

### Treatment

Immediately after the diagnosis of cerebral arteriopathy, 24 (92.3%) subjects were started with antiplatelet therapy. Twenty-one subjects underwent a surgical revascularization (21/26, 80.8%), including indirect bypasses in 12 cases (12/26, 57.1%) and combined direct and indirect procedures in 9 subjects (9/26, 42.9%). In particular, in the combined approaches, STA-MCA + EMS + dural inversion was performed in 6 patients, STA-MCA + EMS was performed in 2 patients, and STA-MCA e EMS left + EDAMPS right in 1 patient. On the other side, in the indirect approaches, EDAS/EDAMPS was performed in 3 cases, EDAS in 3 patients, EDAMPS in 3 patients, EDAPS in 1 patient, burr hole (other center) in 1 case, and EDAS/EMS in 1 patient. Among the 5 subjects who did not undergo surgery (5/26, 19,2%), 3 patients refused surgery. The others were not operated because they had no symptoms and because there was no progression of stenosis/hypoperfusion at follow-up.

The patients who underwent indirect bypass (*n* = 12) received acetylsalicylate therapy (3–5 mg/kg/day, up to a maximum of 75–80 mg/day, according to Scott et al.), which was regularly stopped 5 days before surgery and replaced with low molecular weight heparin (LMWH) prophylaxis at 100 UI/kg [20]. The acetylsalicylate therapy was restarted 48 h after surgery. The patients who underwent combined bypass (*n* = 9) received acetylsalicylate therapy, which was regularly stopped 24 h before surgery and replaced 24/48 h after surgery.

### Outcome

At the time of diagnosis of arteriopathy, the cognitive deficit (pathologic Wechsler Intelligence Scale for Children (WISC)) was present in 10 patients (10/26, 38.5%). The patients with pathologic WISC who refused the surgery (3/10) experienced disease progression (ischemic stroke ± seizure episodes) at 1/2-year follow-up. The remaining 7 patients with pathologic WISC after bypass surgery showed radiological and clinical evidence of stability (no stroke, hemorrhage, cognitive deterioration, or progression stenosis) at 1/2 year of follow-up.

The patients without cognitive deficit at the time of diagnosis (normal WISC) were 16 (61.5%). Cognitive deterioration at 1 year of follow-up was observed in 1 patient (1/16) who underwent previously EDAS/EDAMPS bilateral procedure. Progression stenosis at 1 year of follow-up occurred in 1 patient (1/16) who underwent previously STA-MCA + EMS + dural inversion unilateral procedure. The remaining 14 patients with normal WISC at the time of diagnosis, no stroke, hemorrhage, cognitive deterioration, or progression stenosis occurred at 1 year of follow-up (4 of these remaining 14 patients did not undergo surgery).

During the follow-up, 10 patients developed a brain tumor (7 optic pathway gliomas (OPG), 2 extra OPG, 1 OPG with extra-OPG). Five patients received chemotherapy. Three patients underwent radiotherapy and surgery tumor was performed in 4 cases.

Through logistic regression analysis, pathological WISC at onset was identified as being associated with symptom improvement at 1-year follow up (*p* = 0.006) (Table 2).

**Table 2:** Factors associated with improvement in symptoms at 1 year

		No symptom improvement	Symptom improvement	OR (95%CI); *p*
Sex	Male	4 (36.4%)	7 (63.6%)	M vs F: 2.63 (0.53–13.07); 0.24
	Female	9 (60.0%)	6 (40.0%)	
Age (y)		9.6 ± 5.20	6.4 ± 4.56	0.86 (0.71–1.04); 0.13
Cognitive status at onset (pathologic WISC)	Normal	12 (75.0%)	4 (25.0%)	27.00 (2.56–284.70); 0.006
	Pathologic	1 (10.0%)	9 (90.0%)	
Bypass	No	3 (60.0%)	2 (40.0%)	1.65 (0.23–11.99); 0.62
	Yes	10 (47.6%)	11 (52.4%)	
MCA	No	1 (33.3%)	2 (66.7%)	0.46 (0.04–5.79); 0.55
	Yes	12 (52.2%)	11 (47.8%)	
ACA	No	11 (57.9%)	8 (42.1%)	3.44 (0.53–22.43); 0.20
	Yes	2 (28.6%)	5 (71.4%)	
ICA	No	7 (53.8%)	6 (46.2%)	1.36 (0.29–6.36); 0.70
	Yes	6 (46.2%)	7 (53.8%)	
PCA	No	11 (61.1%)	7 (38.9%)	4.71 (0.73–30.28); 0.10
	Yes	2 (25.0%)	6 (75.0%)	
Tumor	No	8 (50.0%)	8 (50.0%)	1.00 (0.21–4.86); 0.99
	Yes	5 (50.0%)	5 (50.0%)	
Associated vasculopathies	No	12 (50.0%)	12 (50.0%)	1.00 (0.06–17.90); 0.99
	Yes	1 (50.0%)	1 (50.0%)	
Perfusion improved	No	3 (50.0%)	3 (50.0%)	1.00 (0.16–6.35); 0.99
	Yes	9 (50.0%)	9 (50.0%)	
MMD progression	No	5 (55.6%)	4 (44.4%)	1.61 (0.31–8.32); 0.57
	Yes	7 (43.8%)	9 (56.3%)	

*WISC* Wechsler Intelligence Scale for Children, *MCA* middle cerebral artery, *ACA* anterior cerebral artery, *ICA* internal carotid artery, *PCA* posterior cerebral artery

No significant differences were found in long-term neurocognitive outcome and stroke rate between combined and indirect bypass (*p* > 0.05) (Table 3).

**Table 3:** One-year outcome

		No symptoms	Symptoms	OR (95%CI); *p*
Type of surgery	Indirect	10 (55.6%)	2 (66.7%)	0.63 (0.05–8.20); 0.72
	Combined	8 (44.4%)	1 (33.3%)	

## Discussion

Children with NF1 manifest a wide spectrum of cerebrovascular abnormalities with an overall prevalence of approximately 2.5%, including narrowed or ectasic vessels, vascular stenoses, aneurysms, and moyamoya framework [18, 2]. On other hand, moyamoya disease (MMD) is characterized by bilateral steno-occlusive changes at the terminal portion of the ICA and an abnormal vascular network at the base of the brain. Patients with MMD present with the characteristic moyamoya vasculopathy in the absence of associated risk factor. The etiology is unknown. MMD with unilateral involvement and no associated underlying disease has been defined probable MMD [19]. If associated risk factors are present, the vasculopathy is defined MMS. This definition includes unilateral or bilateral cerebrovascular lesions in association with several underlying medical conditions, especially NF1, sickle cell disease, and Down syndrome [20]. In such cases, MMS presents an extreme variability in terms of epidemiology, disease course, age of onset, progression, and complication rate. This picture is further complicated by the frequently unpredictable natural history of the underlying disorder and by the additional issues related to medical treatment. In the light of these considerations, a growing need for data about moyamoya dysplasia-associated NF1 is emerging in order to delineate indications for revascularization surgery, especially in asymptomatic or mildly symptomatic cases.

### Epidemiology

MMD has a higher incidence than MMS (0.35/100,000 versus 0.11/100,000, respectively) whereas the gender distribution (characterized by female predominance) is similar. Also similar to the non-NF1 population, there was a female preponderance in this series (15 females, 11 males). MMD is characterized by bilateral progressive stenosis or occlusion of the basal cerebral arteries, associated with abnormal net-like vessels at the base of the brain. On the other hand, a unilateral involvement may occur in MMS, in the literature in these patients, the vasculopathy is usually unilateral at the beginning and involves anterior vascular territories [21, 22]. In our study, we noted that in 13 cases the vascular involvement was unilateral and we showed that the vascular unilateral involvement remained unilateral over time and there was no tendency to spread to the other side.

According to literature review, based on 181 cases with NF1 and MMS between 1995 and 2015, the age at diagnosis of MMS in patients with NF1 varies dramatically among different series, with a median age ranging from 5.2 years [23] to 11.7 years [24]. In our series of 26 patients, the mean age at diagnosis was 8.0 ± 5.06. Of interest also in the study by Rea et al. was the presence of optic glioma in 13 of the 17 (76%) patients with arteriopathy, the majority having extensive tumors [23]. In the present study, 7 patients had optic glioma, making the association of vasculopathy and optic glioma significant and rife for further study.

### Clinical manifestations

Headache and focal neurological deficits were the most frequent presenting symptom in our series. All the patients experienced a gradual resolution of this symptom after the surgical treatment. In this regard, it has been hypothesized that headache could result from the stimulation of dural nociceptors by the compensatory dilatation of meningeal and leptomeningeal collateral vessels occurring in response to cerebral hypoperfusion [20]. In contrast, occasional diagnoses without symptoms were 12 cases (46.2% of patients). This is probably due to the precise follow-up performed: every year children diagnosed with NF1 undergo a perfusion magnetic resonance imaging (p-MRI) and magnetic resonance angiography (MRA); in this way, an acute event such as severe ischemia can be prevented (Supplementary Fig. 1).

### Radiology

Radiographically, the MRI appearance of NF1-related and non-NF1 moyamoya is indistinguishable, with both groups showing FLAIR hyperintensity (“ivy sign”) in underperfused cortex, a characteristic narrowing of the parent vessels in the anterior circulation of the brain, and in later stages evidence of collateral formation and strokes in the same vascular distribution [25]. At follow-up, 18 cases were shown perfusion improved (18/24, 75.0%).

### Natural history of the underlying disease

Children with NF1 who develop moyamoya are 8 times more likely to be asymptomatic at the time of initial diagnosis than children with moyamoya who do not have NF1—presumably secondary to the surveillance scanning performed in the NF1 population, thus increasing the probability of discovering moyamoya as an incidental finding [26]. In fact, a large proportion of our patients were also asymptomatic at the time of initial diagnosis.

The development of MMS is a well-known complication of radiation therapy in patients with brain tumors [27]. It has been shown that children with NF1 treated with radiation therapy are at increased risk for developing MMS and this risk is proportional to the radiation dose to the circle of Willis [26]*.* Moreover, children with NF1 are potentially at increased risk for neurocognitive compromise after treatment with radiation [28]. In addition, in the literature, the perioperative stroke and complication rates were higher in patients who were treated with radiation despite similar rates of disease progression in patients with NF1 and moyamoya who did not undergo radiation treatment, suggesting that other factors independent of moyamoya may be contributing to the higher risk in this population. Of note, the administration of selumetinib, a MAP kinase (MEK) 1/2 inhibitor which was revealed to be helpful to treat NF1-associated tumors such as plexiform neurofibromas and optic way gliomas, has been recently associated with revascularization failure in NF1-related MMS [29]. In contrast, in our study, we demonstrated that complication rates in patients treated with cranial irradiation are low. For these reasons, it is necessary to valuate surgical revascularization in patients treated with cranial irradiation, even when the vascular disease is asymptomatic. In this way, we can achieve a good long-term outcome with improved intellectual function and prevention of stroke.

Approximately 30% of patients with MMD have presented with involvement of the posterior circulation, mainly the posterior cerebral artery (PCA) [30]. In MMS as NF1 the natural history is different because usually the worsening of posterior circle does not follow the anterior. The onset is dependent territory and the reason is unclear. Common is the symptomatology where headache is the most common symptom and ipsilateral to posterior cerebral artery stenosis or visual field oscillation hypoperfusion.

After completion of the surgeries, patients in this study were followed up regularly in the outpatient clinic with the following imaging studies: p-MRI and MRA at 3–6 months after the last surgery, p-MRI and MRA every year thereafter.

### Cognitive impairment

The cognitive impairment is another important aspect to consider in patients with MMS. Indeed, on the basis of the natural history of NF1, approximately 80% of children develop deficits in one or more cognitive functions [12]. In addition, an increased frequency of attention deficit hyperactivity disorder (ADHD) and autistic spectrum disorders has also been observed in patients with NF1 and other RASopathies [31, 32]. Studies on cognitive ability in these patients suggest that the prevalence of intellectual impairment (IQ < 70) is about 20% [33]. The data on cognitive impairment in patients with MMS are still inconsistent and unsuitable to draw conclusions about the outcome after surgery or medical therapy alone. However, we can show that in post-operative of our series, 20 out 21 cases showed an intellectual stability quantifiable with the WISC scale at 1–2-5 year of follow-up. In this study, pathological WISC at onset was identified associated with symptom improvement at 1-year follow-up. This could play a role in the correct selection of patients for surgery. On the other hand, in order to have sufficient statistical power for other data, it is necessary to have a larger sample size to detect smaller effects.

### Timing of surgery

The timing of diagnosis relative to the timing of surgery in the NF1-moyamoya population is important. As noted above, many of the children in this series were diagnosed with moyamoya while asymptomatic than comparable populations in non-NF1 moyamoya, presumably related to the screening studies performed for other NF1-related pathology. There may be a lag of several months to years between the radiologic manifestations and the development of ischemic symptoms or signs secondary to NF1-associated cerebral vasculopathy [34].

It has been established that clinical status at the time of moyamoya diagnosis and surgery is the most important determinant of overall outcome [35]. As such, there is a unique opportunity with these children to substantively reduce risk by treating them with early surgical intervention, prior to the onset of a debilitating stroke. This is also supported by the observations that many children developed clinical symptoms over time and that radiographic progression occurred in 40% of patients; moreover, children who exhibited clinical symptoms preoperatively had the highest risks of perioperative complication [25]. While clinicians need to exercise caution in confirming the diagnosis before committing a patient to surgery and while the risks and benefits of each case must be determined individually, there is compelling evidence that reinforces the benefits of early diagnosis and justifies a proactive stance favoring early surgical revascularization. In our series, we noted that there was only a clinical worsening case in cognitive outcome. We noted that in 20 cases there were no post-operative complications such as seizures, progression stenosis, or ischemia at 1–2 follow-up.

Revascularization prior to significant damage has been shown to improve cognition and quality of life, prevent neurocognitive decline if performed early, and modulate the natural history of the disease [13]. In fact, clinical evidence supports the crucial role of early diagnosis and surgical revascularization to achieve a good long-term outcome with improved intellectual function and stroke prevention in these patients [36–38].

Some of these patients are affected by slow-growing tumors and need chemotherapy and/or RT, which both can worsen the vasculopathy. Considering the good prognosis of the associated tumors (e.g., OPGs) and that a delayed treatment usually does not affect the outcome, a prophylactic surgical revascularization should be considered in patients that must receive chemotherapy or RT and with initial cerebrovascular disease [27]. Another aspect to be emphasized is that 3 cases of patients who refused surgery had a clinical worsening with episodes of ischemic stroke or convulsions in the follow-up.

### Surgical considerations

Direct revascularization carries the risk of hyperperfusion syndrome, which results from a sudden increase in cerebral blood flow; the incidence is as high as 28% and is seen most often in patients with extensive preoperative ischemia [39, 40]. Hyperperfusion syndrome can result in a temporary clinical picture that includes sensorimotor loss, aphasia, and dysarthria [41]. Lastly, the procedure is challenging in the pediatric population because of the patients’ smaller, more delicate cerebral vasculature [21].

Indirect techniques rely on tissue supplied by the external carotid artery, which is placed on the brain surface and promotes angiogenesis. This approach generally requires less operative time since it is both technically less challenging and less invasive [42]. Importantly, the indirect approaches do not require temporary occlusion of MCA branches, which is required in the STA-MCA bypass [16]. As such, indirect approaches have a better safety profile than direct procedures and are preferred in children and adults with other medical comorbidities [43]. However, in stark contrast to children, collaterals only form in approximately half of the adult patients following the indirect procedures [44]. Even then, collaterals take months to mature, while the direct procedures provide robust reperfusion immediately [45]. Combined procedures have also been employed by some surgeons who have postulated that the technique reduces the incidence of repeat bleeding; however, there is little evidence to show if the outcomes are actually improved or potentially worsened [46]. Thus, the literature does not present a clearly superior revascularization technique in the surgical management of MMD/MMS. Thus, our results currently offer the best comparison of the indirect and combined approaches. In our series, regarding the choice of the best surgical technique, no significant differences were found in long-term neurocognitive outcome and stroke rate between combined and indirect bypass. Additionally, reintervention was not necessary in any case.

One of the distinct advantages of the indirect techniques is the ability to revascularize the anterior and posterior cerebral artery territories in addition to the middle cerebral artery territory, which is primarily targeted in the direct technique. This is especially important in the pediatric population who may have long-term neurologic dysfunction secondary to widespread hypoperfusion in the anterior, middle, and posterior cerebral artery territories. The process of revascularization and collateralization of the hypoperfused vascular bed is typically slow after the indirect techniques and may be associated with continued clinical symptoms for a period of usually a few months after the surgery. The reduced operative time and invasiveness, as well as the relative ease of the indirect procedures, may contribute to the well-documented decrease in adverse effects. Moreover, it is also possible that the risk of perioperative stroke from the delayed formation of collaterals following indirect procedures may be overstated. Our study noted that pediatric patients tolerated the combined procedure well and did not suffer from an increased rate of adverse events as compared with adult patients undergoing the direct procedure. Two procedures were determined to have equal efficacy; it stands to reason that the choice would be the less invasive approach.

According to the good clinical and radiological results achieved by the surgical revascularization procedures performed under the Tokyo Daigaku (The University of Tokyo) protocol (TODAI protocol) in adult and pediatric patients with MMD (EDAS combined with EMS in patients 9 years old or younger, combined direct STA-MCA anastomosis and EMS in patients 10 years old or older) [47], we believe that EDAS combined with EMS should be considered an essential component of the surgical revascularization in children.

### Hemodynamic status

Besides the importance of studying hemodynamic status, investigation of the collateral blood supply plays a pivotal role in evaluating a bypass indication since collateral blood flow influences perfusion and hemodynamics [48]. The status of collateral circulation can be assessed with transcranial Doppler ultrasonography, CTA/MRA, and digital subtraction angiography. However, none of these modalities provide quantitative flow (ml/min) values. Therefore, quantitative 2D phase-contrast image analysis segments (NOVA software package) allow assessment of direction of blood flow and approximation of flow velocity [49].

Another tool would be the blood oxygen level-dependent (BOLD) MR imaging, a widely used technique for the noninvasive imaging of dynamic changes in CBF at the local and global levels [50]. It can also be used to evaluate surgery in patients with an intermediate stage of disease (modified Suzuki stage II or III) and to follow patients after revascularization [51, 52]. Comprehensively, BOLD MR provides a visual representation of ischemic risk and impending tissue demise. NOVA software and BOLD MR imaging are already used for example in the acute phase in the management of stroke due to large vessel occlusion of the anterior circulation to better understand collateral circulation, as tool to decide if surgical revascularization would be a useful procedure [53].

### Perfusion in children with NF1 without MMS

Since abnormal perfusion is expected in patients with MMS, we specifically targeted patients with NF-1 without this feature. Yeom et al. demonstrated significantly lower CBF in patients with NF-1 compared with control subjects, occurring most prominently in the posterior circulation and the border zones of the middle and posterior cerebral arteries [54]. To our knowledge, this is an important study to report perfusion abnormalities in children with NF-1 in the absence of prior strokes or underlying MMS.

On the other hand, cognitive complications are relatively common in NF-1, ranging from 30 to 65%, and include developmental delay, learning disabilities, attention deficit disorder, and headaches [12]. If the relationship between NF-1 arteriopathy and cognitive involvement was clear, surgical revascularization could also play a role in NF-1 patients with abnormal perfusion, in the absence of a true moyamoya diagnosis.

## Conclusion

In this study, we reviewed our case series of patients with moyamoya-associated NF1 and analyzed clinical, neuroimaging, and surgical data to investigate the role of surgical revascularization as a relevant procedure to prevent ischemic manifestations and neurocognitive decline in pediatric patients. Our study has some limitations. Indeed, it was not feasible to conduct a comprehensive follow-up for up to 5 years on all patients. Additionally, it is important to note that this study might lack sufficient statistical power, suggesting the need for a larger sample size to detect statistically significant results when a real effect is present. A larger sample size would enhance the reliability and robustness of the study’s findings.

Despite these limitations, our observations may have significant implications in the management of pediatric patients with moyamoya-associated NF1. In patients diagnosed with NF1, we suggest yearly radiological follow-up through p-MRI e MRA, in order to prevent an acute event. In case of mild symptoms such as headache or hypoperfusion or stenosis progression, we recommend mono- or bilateral bypass surgery, depending on the vascular involvement and the clinic. We suggest considering prophylactic surgical revascularization in MMS patients with a good overall prognosis and a cerebrovascular disease in early stages. In our opinion, this strategy could help prevent the deterioration of the cognitive function due to the progression of MMS. This protective surgery could offer the opportunity to preserve the quality of life of these patients, without any significant detrimental impact on the outcome. In addition, in our series, regarding the choice of the best surgical technique, no significant differences were found in long-term neurocognitive outcome and stroke rate between combined and indirect bypass.

The decision to propose surgical cerebral revascularization may be difficult because many distinctive aspects have to be considered such as noncerebral vasculopathy or in case of tumors in close proximity to the circle of Willis. Further multicentric studies are needed to shed some light on the treatment outcome in these complex patients due to the limited data available.

### Supplementary Information

Below is the link to the electronic supplementary material.

Supplementary file1 (DOCX 24 KB)

## Data Availability

No datasets were generated or analyzed during the current study.
