# Supplementary material for: Surgical revascularization as a procedure to prevent neurological complications in children with moyamoya syndrome associated with neurofibromatosis I: a single institution case series
Source: Childs Nerv Syst. 2024 Feb 6;40(6):1731–41. doi: 10.1007/s00381-024-06304-z (PMC11111570; doi:10.1007/s00381-024-06304-z)
Supplement: Supplementary file 1 — Supplementary file1 (DOCX 24 KB) [file 381_2024_6304_MOESM1_ESM.docx]

**Supplementary Figure 1 *Flow-chart Gaslini Institute***

p-MRI= perfusione-MRI; MRA= MR angiography
